# Ovarian mesonephric-like adenocarcinoma arising in serous borderline tumor: a case report with complex morphological and molecular analysis

**DOI:** 10.1186/s13000-020-01012-z

**Published:** 2020-07-21

**Authors:** Pavel Dundr, Mária Gregová, Kristýna Němejcová, Michaela Bártů, Nikola Hájková, Jan Hojný, Ivana Stružinská, Daniela Fischerová

**Affiliations:** 1 Institute of Pathology, First Faculty of Medicine, Charles University and General University Hospital in Prague, Studnickova 2, 12800 Prague 2, Czech Republic; 2 Gynecologic Oncology Center, Department of Obstetrics and Gynecology, First Faculty of Medicine, Charles University and General University Hospital in Prague, Apolinarska 18, 12808 Prague 2, Czech Republic

**Keywords:** Mesonephric-like adenocarcinoma, Serous borderline tumor, Ovary, Case report

## Abstract

**Background:**

Mesonephric-like adenocarcinoma (M-LAC) is a rare, recently described tumor occurring in the uterine corpus and ovary, which shares the same morphological and immunohistochemical features with the more common mesonephric adenocarcinoma (MAC), which mostly arises the uterine cervix. Despite the similarities between these tumors, the histogenesis of M-LAC is still disputable.

**Case presentation:**

Sixty-one-year-old woman presented with an advanced tumor of the left ovary with intraabdominal spread and liver metastases. After receiving 5 cycles of neoadjuvant chemotherapy, she underwent a hysterectomy with bilateral salpingo-oophorectomy, and resection of the liver metastasis, omentum, and appendix. Histologically, the ovarian tumor consisted of two components, whose morphology and immunohistochemical results were typical of either a serous borderline tumor (immunohistochemical positivity for PAX8, WT1, ER and PR) or a mesonephric-like carcinoma (immunohistochemical positivity for PAX8, TTF1 and GATA3). Only the component of the mesonephric-like adenocarcinoma metastasized to the omentum and liver. A molecular analysis with a panel of 271 genes (size 1020 kbp) was performed separately on samples from the borderline tumor, primary ovarian mesonephric-like adenocarcinoma, and liver metastasis. The results showed the clonal origin of all samples, which shared the same *KRAS* (NM_004985.3:c.34G > T, p.(G12C)) and *PIK3CA* (NM_006218.2:c.1633G > A, p.(E545K)) somatic mutations. Moreover, in the sample from the primary mesonephric-like carcinoma and its liver metastasis a likely pathogenic somatic *MYCN* mutation (NM_005378.4:c.131C > T, p.(P44L) was found. In all samples, the deletion of exons 9–10 in the *CHEK2* gene was present, which is in concordance with the previously performed genetic testing of the blood specimen which revealed the hereditary *CHEK2* mutation in this patient.

**Conclusions:**

Our result support the theory that at least some mesonephric-like ovarian adenocarcinomas are of Müllerian origin. The serous borderline tumor seems to be a precursor of mesonephric-like adenocarcinoma, which has been proven in our case by both tumors sharing the same mutations, and the presence of cumulative molecular aberrations in the mesonephric-like adenocarcinoma.

## Background

Mesonephric-like adenocarcinomas (M-LAC) are rare, recently described tumors occurring in the uterine corpus and ovary, which share the same morphological and immunohistochemical features with more common mesonephric adenocarcinoma (MAC), most commonly arising in the uterine cervix [1–5]. Despite the similarities between these tumors, the histogenesis of M-LAC is still disputable, as this type of tumor occurs outside the anatomical areas in which mesonephric remnants/hyperplasia may normally occur. Recently, two cases of ovarian M-LAC arising in a serous borderline tumor (S-BTO) and a low grade serous carcinoma (LGSC) have been described. In both of these cases, the Müllerian tumors shared the same mutations as M-LAC, which proved their clonal origin [6, 7]. Based on this finding, the authors suggest that at least some of the M-LACs may be of Müllerian origin. In our report, we present another case of M-LAC arising in association with a S-BTO, with molecular evidence of the clonal origin of both tumors sharing the same *KRAS* and *PIK3CA* mutations.

## Case presentation

Sixty-one-year-old female was referred in June 2019 to our Gynecologic Oncology Centre for interval debulking surgery (IDS) after previous administration of 5 cycles of neoadjuvant chemotherapy (NAC) for a primarily non-resectable left ovarian tumor FIGO (the International Federation of Gynecology and Obstetrics) classification [8] stage IV (presence of liver metastases). After NAC (paclitaxel plus carboplatin) the computed tomography findings revealed a partial regression tumor response according to RECIST (The Response Evaluation Criteria in Solid Tumors) criteria [9]. The size of the primary ovarian tumor mass and peritoneal carcinomatosis was reduced. However, the intraparenchymatous liver metastases remained unchanged in number and size. In July 2019, the patient underwent optimal IDS with no visible residual disease at the end of surgery. The surgery consisted of a hysterectomy with bilateral salpingo-oophorectomy, resection of liver metastases and the diaphragm, total omentectomy, appendectomy and a resection of an umbilical metastasis. After the surgery, the patient received 3 additional cycles of adjuvant chemotherapy (PTX/CBDCA), with the addition of bevacizumab from the second post-operative cycle for a period of 12 months. Recently, she underwent her 11th cycle of bevacizumab and is currently showing signs of complete clinical remission.

### Pathologic findings

The left ovary was partly solid, partly cystic, and measured 35 × 30 × 20 mm. The left Fallopian tube was encased in adhesions together with the ovary. The uterus, right ovary, and the other fallopian tube were grossly normal. The omentum, measuring 450 × 180 × 10–90 mm, showed multiple small nodules (up to 10 mm in diameter) and one larger nodule (90 mm in diameter). The liver excision of 80 × 50 × 25 mm showed a few nodules up to 25 mm in the largest dimension.

**Fig. 1:**
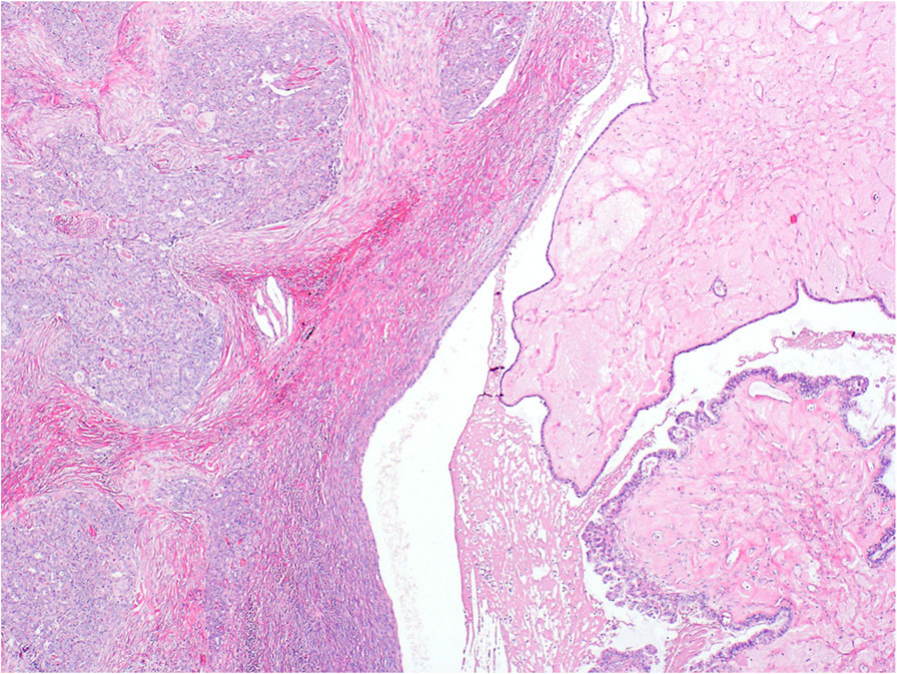
Low power view showing serous borderline tumor (right) and mesonephric-like adenocarcinoma (left) (HE, 40x)

**Fig. 2:**
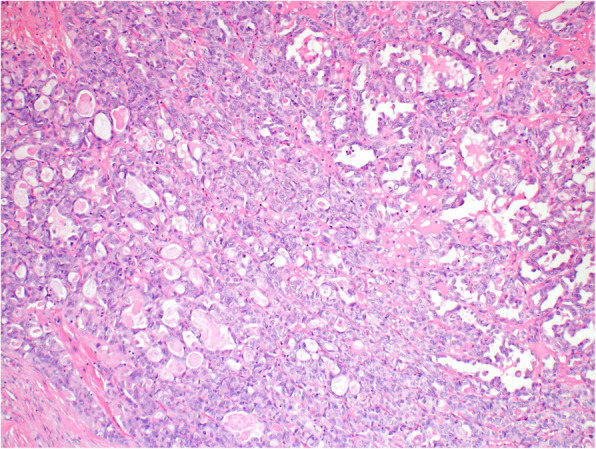
Mesonephric-like adenocarcinoma showing a predominantly tubular pattern with eosinophilic intraluminal secretions (HE, 100x)

**Fig. 3:**
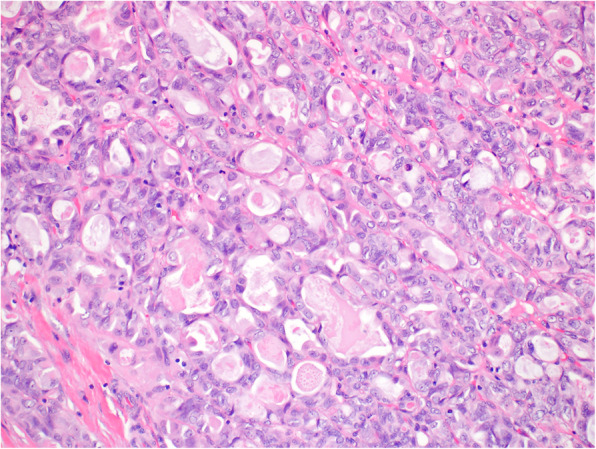
Higher magnification of mesonephric-like adenocarcinoma, showing tumor cells with vesicular nuclei and small nucleoli. Note the few cells with hobnail nuclei (HE, 200x)

Histologically, in the left ovary there were structures of a S-BTO with typical features (Fig. 1). The examination also revealed that in close proximity to this tumor there were structures of invasive carcinoma, with mostly tubular, tubulocystic, and sporadically solid architecture. In the lumen of some of the tubular formations a dense eosinophilic material could be found (Fig. 2). Tumor cells were medium in size, with predominantly vesicular nuclei with small nucleoli (Fig. 3). Sporadic hobnail cells were present. The mitotic count was up to 4 mitoses /10 HPFs. The cytoplasm was predominantly eosinophilic. The structures of invasive carcinoma were present on the surface of the ovary and the serosa of the left fallopian tube. Metastases were found in the omentum and liver. The S-BTO was confined to the left ovary, without any sign of metastatic spread.

**Fig. 4:**
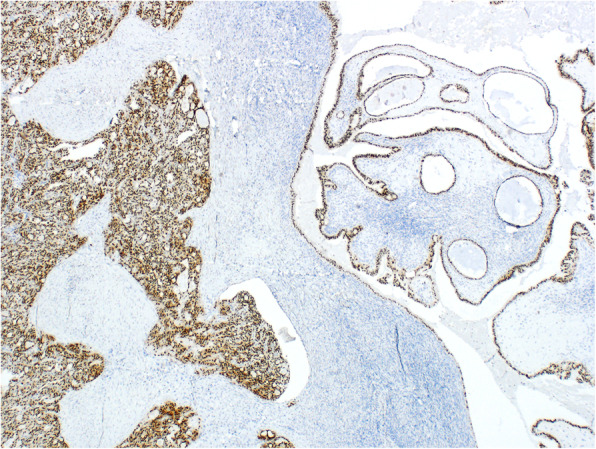
Immunohistochemical finding showing PAX8 positivity of the tumor cells (40x). Note the positivity in both the serous borderline tumor (right) and mesonephric-like adenocarcinoma (left)

**Fig. 5:**
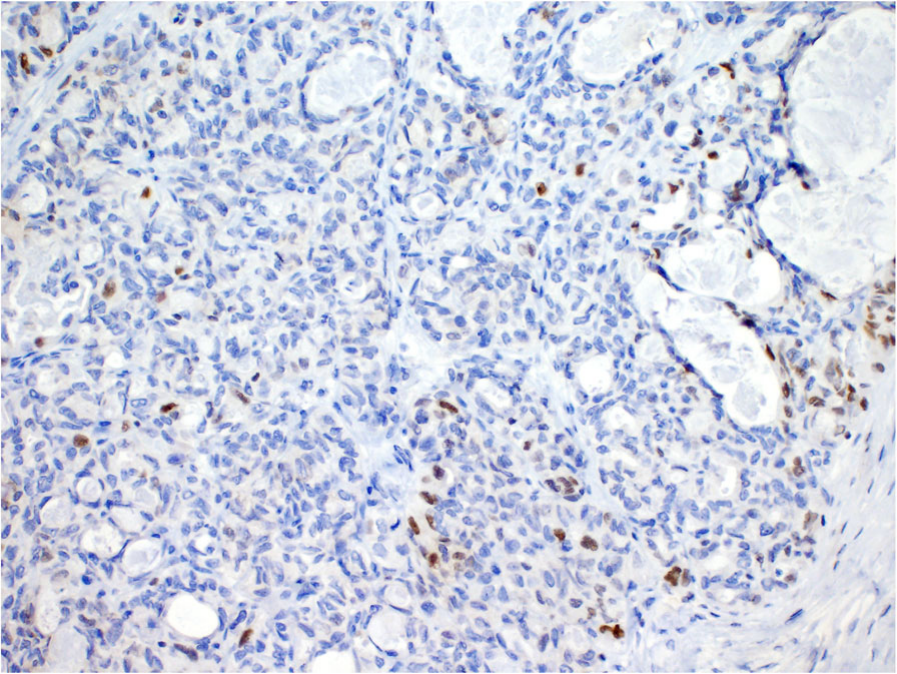
Mesonephric-like adenocarcinoma showing focal GATA3 staining (200x)

**Fig. 6:**
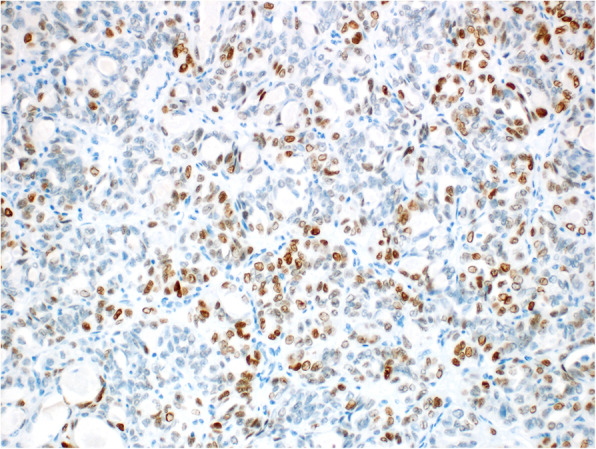
Mesonephric-like adenocarcinoma showing focal TTF1 staining (200x)

Immunohistochemically, the two tumor types showed different results. The S-BTO showed diffuse positivity for PAX8 and estrogen receptors. Progesterone receptors were expressed in approximately 50% of the S-BTO tumor cells. The structures of invasive carcinoma showed diffuse positivity for PAX8 (Fig. 4). Up to 20% of the tumor cells were also positive for GATA3 (Fig. 5). Most of the tumor cells (approximately 70%) also showed positiveTTF1 expression of variable intensity (Fig. 6). Focally, there was a luminal positivity of CD10. The p53 showed a wild-type expression pattern. All of the other examined markers including WT1, estrogen receptor, progesterone receptor, HNF1B, calretinin, and inhibin were negative. Based on the morphology and the immunohistochemical profile, the invasive carcinoma was classified as an M-LAC.

The DNA from the FFPE samples was prepared and analyzed according to our standardized protocol [10]. Libraries from the isolated DNA were prepared using the SeqCap custom hybridization probes (1020 kbp custom panel of 271 genes, NimbleGen, Roche) and sequenced by the NextSeq 500 instrument using the NextSeq 500/550 Mid Output Kit v2.5 (150 Cycles) separately on samples from the borderline tumor, invasive carcinoma, and liver metastasis. The results showed the clonal origin of all samples, which shared the same *KRAS* (NM_004985.3:c.34G > T, p.(G12C)) and *PIK3CA* (NM_006218.2:c.1633G > A,p.(E545K)) mutation. Moreover, in the sample from the primary invasive carcinoma and its liver metastasis, a likely pathogenic *MYCN* mutation (NM_005378.4:c.131C > T, p.(P44L) was found. In all samples, the deletion of exon 9–10 in the *CHEK2* gene was also discovered, in concordance with the already known hereditary *CHEK2* mutation carried by this patient.

## Discussion

MACs of the female genital tract are rare tumors, usually arising in the uterine cervix and vagina [2, 3, 11]. Histologically, these tumors are characterized mostly by a mixture of heterogeneous architectural patterns. The most common is tubular or glandular with the formation of small glands, with a common presence of eosinophilic intraluminal colloid-like material. However, other patterns may occur, including solid (with possible spindled morphology), slit-like, papillary, glomeruloid, sex cord-like, retiform, and glandular with the formation of medium-sized or large glands [3, 12–14]. The tumor cells may be flattened, cuboidal or columnar, with usually scant pale eosinophilic or, rarely, clear cytoplasm. The nuclei are usually vesicular, commonly resembling those of papillary thyroid carcinoma, and may show grooving, irregular nuclear membranes, and in some cases nuclear pseudoinclusions. Hobnail cells can be present as well. Immunohistochemically, these tumors may be positive for CD10, GATA3, TTF1, HNF1B, PAX2 and PAX8 [12, 13, 15]. The p16 expression is patchy in most cases, and p53 expression is of the wild-type. WT1, estrogen and progesterone receptors are negative, although a focal expression of the estrogen receptor has been described in rare cases. On a molecular level, these tumors are characterized by the *KRAS* mutation which occurs in most cases (∼80%) [14, 16]. The *ARID1A* mutation is also common (∼50% of cases), as well as the gain of chromosome 1q detected by copy number analysis (∼80% of cases). Other mutated genes such as *NRAS* (1/16 cases), *ARID1B* (3/16 cases), *BCOR* (2/16 cases), *BCORL1* (2/16 cases), *SMARCA4* (2/16 cases), *TP53* (1/16 cases), and *PTCH2* (4/12 cases) have also been described [14, 16, 17]. A single case report of mutation in *MAC* and *CTNN1B* (occurring together with the KRAS mutation) was reported [11].

Rare tumors with similar morphological and immunohistochemical features have also been described in the uterine corpus and ovary [1, 14]. However, the mesonephric origin of these tumors has not yet been proven as these tumors arise in anatomical areas not associated with mesonephric remnants. In a recent study of 12 cases (7 endometrial and 5 ovarian) none of the endometrial cases were associated with mesonephric remnants in the uterine cervix, and all arose in endometrium. Three of the five ovarian cases were associated with endometriosis [18]. Due to these histogenetic uncertainties, the authors of the study prefer the use of term “mesonephric-like adenocarcinoma”. Molecular aberrations occurring in M-LAC are very similar to MAC, with common *KRAS* mutation occurring in ∼90% of cases [1, 4, 16, 17]. However, contrary to MAC, in ∼35% of M-LAC the mutation of *PIK3CA* has been reported, which has not yet been described in MAC. This was also observed in our case, where we detected both *KRAS* and *PIK3CA* mutations. Supporting the assumption of the Müllerian origin of M-LAC, there are two recent reports of ovarian cases associated with Müllerian tumors (in both cases with S-BTO and LGSC), with molecular analysis confirming the same clonal origin of these tumors [6, 7]. Based on these results, at least some M-LACs of the ovary are Müllerian in origin and the term “mesonephric-like adenocarcinoma” therefore seems to be appropriate. In the first case, the authors described a tumor consisting of LGSC and M-LAC areas [6]. Both tumor components had metastasized. The LGSC arose in association with a serous borderline tumor. All components (S-BTO, LGSC and M-LAC) were proven to be clonal in origin and shared the same mutation in the *NRAS* gene p.(Q61R). Moreover, additional aberrations were detected in the LGSC metastasis (*KDM5A*, *STAG2*) and the M-LAC (primary – *BCOR*, *AMER1*, metastasis – *BCOR*). The second case describes an M-LAC arising in an S-BTO in a patient with bilateral S-BTO and LGSC, with invasive implants [7]. All components (serous BTO, LGSC and M-LAC) were proven to be clonal in origin and shared the same *KRAS* mutation p.(G12D). In our case, the serous BTO and M-LAC were also confirmed to be clonal in origin and shared the same *KRAS* p.(G12C) and *PIK3CA* p.(E545K) mutations. In addition, in the M-LAC the mutation of the *MYCN* gene was found.

The differential diagnosis of M-LAC includes other epithelial tumors, especially endometrioid carcinoma (EC) [1]. The distinction between M-LAC and EC may be difficult and should be based on morphological and immunohistochemical features. M-LAC is characterized by common heterogeneity of architectural patterns. These patterns are not specific, but if tubular areas with intraluminal eosinophilic secretions are present, the suspicion of M-LAC is high. Cytological features can be helpful as well, because nuclei with vesicular chromatin and nuclear grooves typical for M-LAC are not a characteristic feature of EC. Immunohistochemically, M-LAC is characterized by GATA-3 and TTF1 expression, which is rare in EC. On the contrary, EC is characterized by common expression of estrogen and progesterone receptors, which are usually absent in M-LAC [1].

In conclusion, we report a case of ovarian M-LAC arising in a S-BTO. The clonal origin of both components suggests that S-BTO may be a precursor of M-LAC. This is in concordance with two previously reported ovarian M-LAC cases associated with Müllerian tumors [6, 7]. Our findings, and the results of the two previous reports, suggest that at least some ovarian M-LACs are of Müllerian origin and the term “mesonephric-like adenocarcinoma” instead of “mesonephric adenocarcinoma” seems to be appropriate. However, M-LACs of the uterine corpus and ovary share the morphology, immunophenotype, molecular aberrations (with the exception of *PIK3CA* mutation) and, based on the limited experience, also the aggressive behavior with the more common MAC of the uterine cervix (and cases classified as MAC of the uterine corpus). Despite the different origin of these tumors (Müllerian vs. mesonephric), the lineage of “mesonephric-like adenocarcinoma” differentiation seems to be identical to MAC, as evident by the sharing of all mesonephric features including molecular aberrations, which can simply reflect the multipotential differentiation capacity of Müllerian structures. Based on this, the classification of these tumor as “mesonephric-like” seems to be rather arbitrary and reflective more of the histogenetic origin and primary location of the tumor, than of the morphology, molecular aberrations, and biological potential.

## Data Availability

The datasets generated during the current study are available from the corresponding author on reasonable request.

## Abbreviations

FFPE: Formalin-fixed paraffin-embedded
LGSC: Low grade serous carcinoma
M-LAC: Mesonephric-like adenocarcinoma
MAC: Mesonephric adenocarcinoma
S-BTO: Serous borderline tumor
